# Comparative assessment of sarcopenia using the JSH, AWGS, and EWGSOP2 criteria and the relationship between sarcopenia, osteoporosis, and osteosarcopenia in patients with liver cirrhosis

**DOI:** 10.1186/s12891-019-2983-4

**Published:** 2019-12-26

**Authors:** Chisato Saeki, Keiko Takano, Tsunekazu Oikawa, Yuma Aoki, Tomoya Kanai, Kazuki Takakura, Masanori Nakano, Yuichi Torisu, Nobuyuki Sasaki, Masahiro Abo, Tomokazu Matsuura, Akihito Tsubota, Masayuki Saruta

**Affiliations:** 1 Division of Gastroenterology and Hepatology, Department of Internal Medicine, The Jikei University School of Medicine, 3-25-8, Nishi-shinbashi, Minato-ku, Tokyo, 105-8461 Japan; 2 Division of Gastroenterology, Department of Internal Medicine, Fuji City General Hospital, Shizuoka, Japan; 3 Department of Rehabilitation Medicine, The Jikei University School of Medicine, Tokyo, Japan; 4 Department of Laboratory Medicine, The Jikei University School of Medicine, Tokyo, Japan; 5 Core Research Facilities, Research Center for Medical Science, The Jikei University School of Medicine, Tokyo, Japan

**Keywords:** Liver cirrhosis, Sarcopenia, Sarcopenia assessment criteria, Osteoporosis, Osteosarcopenia, Vertebral fracture

## Abstract

**Background:**

Sarcopenia and osteoporosis reduce life quality and worsen prognosis in patients with liver cirrhosis (LC). When these two complications coexist, a diagnosis of osteosarcopenia is made. We aimed to investigate the actual situations of sarcopenia, osteoporosis, osteosarcopenia, and vertebral fracture, and to clarify the relationship among these events in patients with LC.

**Methods:**

We describe a cross-sectional study of 142 patients with LC. Sarcopenia was defined according to the Japan Society of Hepatology (JSH) criteria, Asian Working Group for Sarcopenia (AWGS) criteria, and European Working Group on Sarcopenia in Older People (EWGSOP2) criteria. The skeletal muscle mass index (SMI) and handgrip strength were assessed using bioelectrical impedance analysis and a digital grip strength dynamometer, respectively. Bone mineral density (BMD) was measured using dual energy X-ray absorptiometry, and vertebral fracture was evaluated using spinal lateral X-rays. The severity of LC was assessed using the Child-Pugh classification.

**Results:**

Among the 142 patients, the prevalence of sarcopenia was 33.8% (48/142) according to the JSH and AWGS criteria and 28.2% (40/142) according to the EWGSOP2 criteria. The number of patients with osteoporosis, osteosarcopenia, and vertebral fracture was 49 (34.5%), 31 (21.8%), and 41 (28.9%), respectively. Multivariate analysis revealed a close association between sarcopenia and osteoporosis. Osteoporosis was independently associated with sarcopenia [odds ratio (OR) = 3.923, *P* = 0.010]. Conversely, sarcopenia was independently associated with osteoporosis (OR = 5.722, *P* < 0.001). Vertebral fracture occurred most frequently in patients with osteosarcopenia (19/31; 61.3%) and least frequently in those without both sarcopenia and osteoporosis (12/76; 15.8%). The SMI and handgrip strength values were significantly correlated with the BMD of the lumbar spine (*r* = 0.55 and 0.51, respectively; *P* < 0.001 for both), femoral neck, (*r* = 0.67 and 0.62, respectively; *P* < 0.001 for both), and total hip (*r* = 0.67 and 0.61, respectively; *P* < 0.001 for both).

**Conclusions:**

Sarcopenia**,** osteoporosis, osteosarcopenia, and vertebral fracture were highly prevalent and closely associated with one another in patients with LC. Specifically, patients with osteosarcopenia had the highest risk of vertebral fractures. Early diagnosis of these complications is essential for treatment intervention**.**

## Background

Sarcopenia, defined as the loss of skeletal muscle mass and strength, is a common complication of liver cirrhosis (LC) [1]. Originally, the term ‘sarcopenia’ was used to describe age-related decreases in muscle mass [2]. Later, the European Working Group on Sarcopenia in Older People (EWGSOP) defined sarcopenia as a syndrome characterized by decreases in both skeletal muscle mass and strength, which are associated with physical disability, poor quality of life (QOL), and high mortality [3–6]. Furthermore, the Working Group classified sarcopenia into two categories as follows: ‘primary’ (or age-related), when sarcopenia is caused by aging itself; and ‘secondary’, when one or more other causes are evident, such as chronic debilitating disease, including LC. In patients with LC, secondary sarcopenia is an independent predictor of minimal hepatic encephalopathy and poor prognosis [7, 8]. However, with advances in the conservative treatment for LC, patients live to a greater age than before, and are also aging among Japan’s graying population. Therefore, sarcopenia is increasingly prevalent and attracts attention especially in aging patients.

The definition of sarcopenia is changing with time and varies according to which academic society diagnostic criteria are used. In Europe, the EWGSOP established the definition of sarcopenia in 2010 and updated the criteria in 2018 (EWGSOP2) based on accumulated evidence [3, 9]. Asia has a large, rapidly aging population; therefore, the Asian Working Group for Sarcopenia (AWGS) developed the definition of sarcopenia for Asian people in 2014 [10]. These definitions included impaired physical performance and loss of muscle mass and strength in the aged population. Meanwhile, the Japan Society of Hepatology (JSH) proposed sarcopenia criteria for patients with chronic liver disease (CLD) in 2015 [1]. The JSH criteria use identical cut-off values for muscle mass and strength as the AWGS criteria, but the age-related criterion and assessment of physical performance (gait speed) are omitted. Different definitions, depending on which criteria are used (JSH, AWGS, or EWGSOP2), may alter the diagnosis of sarcopenia, resulting in the variation in disease prevalence. Therefore, research to analyze the actual situation based on several sarcopenia criteria is required in order to take preventative measures against sarcopenia in patients with LC.

Osteoporosis, a metabolic bone disorder characterized by compromised bone strength, is a common complication in patients with LC and affects the QOL due to chronic pain and immobility [11]. Reportedly, the prevalence of osteoporosis among patients with LC varies from 12 to 55% in the West [12], whereas the prevalence remains uncertain in Japan. Patients with osteoporosis are susceptible to fractures of bones, such as vertebrae, the femoral neck, and peripheral bones. Specifically, vertebral fracture occurs frequently in patients with LC, and its prevalence ranges from 7 to 35% [11]. However, as vertebral fracture often develops without symptoms, the prevalence of symptomatic and asymptomatic vertebral fracture in Japanese patients remains unclear.

A previous report demonstrated the relationship between sarcopenia and osteoporosis in a Japanese elderly community-based population [13]. The prevalence of sarcopenia among subjects aged ≥60 years was 8.2%, and the cumulative incidence of sarcopenia was 2.0% per year. The prevalence of osteoporosis among patients with sarcopenia was 57.8%, which was significantly higher than those without sarcopenia [13]. On the other hand, the prevalence of osteoporosis among subjects aged ≥60 years was 24.9%, and the prevalence of sarcopenia among those with osteoporosis was 19.1%. Moreover, osteoporosis was an independent predictor of the occurrence of sarcopenia [13]. Sarcopenia and osteoporosis are interrelated and closely linked in terms of common risk factors and biological pathways; therefore, the term ‘osteosarcopenia’ (originating from the term ‘sarco-osteopenia’) was defined as when the two diseases coexist [14, 15]. Patients with osteosarcopenia have a higher risk of falls, fractures, and frailty [14–16].

Therefore, comprehensive assessments and strategies for skeletal muscle and bone disorders, such as sarcopenia and osteoporosis, are essential to improve QOL and morbidity in patients with LC. However, there are few reports evaluating the association between low skeletal muscle mass and strength, osteoporosis, and vertebral fracture in the same individuals with LC. The aim of this study was to investigate the actual situation of sarcopenia according to the JSH, AWGS, and EWGSOP2 criteria and to clarify the relationship between sarcopenia, osteoporosis, osteosarcopenia, and vertebral fractures in Japanese patients with LC.

## Methods

### Study design and patients

This was a cross-sectional study that included 169 consecutive patients who were diagnosed with LC between October 2017 and March 2019 at the Division of Gastroenterology, Department of Internal Medicine, Fuji City General Hospital (Shizuoka, Japan). The inclusion criteria were as follows: (i) presence of LC; (ii) measurement of skeletal muscle mass index (SMI) using the bioelectrical impedance analysis (BIA) method; (iii) measurement of handgrip strength using a grip dynamometer; (iv) measurement of bone mineral density (BMD) using dual energy X-ray absorptiometry (DEXA); and (v) evaluation of vertebral fracture using spinal lateral X-rays. Liver cirrhosis was diagnosed based on laboratory tests, morphological assessment with imaging (ultrasonography, computed tomography, and magnetic resonance), and/or the presence of esophageal or gastric varices confirmed by upper gastrointestinal endoscopy. The severity of LC was estimated by the Child-Pugh classification. Briefly, the Child-Pugh score consists of the following five clinical components: total bilirubin, serum albumin, prothrombin time (PT), degree of ascites, and grade of encephalopathy. Each component is scored from 1 to 3, and the total scores are calculated by summation and are classified into class A (5–6 points), B (7–9 points), and C (10–15 points), with class C being the most severe [17]. The exclusion criteria were as follows: (i) patients with existing refractory ascites; (ii) patients with existing metal in their body; and (iii) patients undergoing hemodialysis, because these conditions may result in the overestimation of skeletal muscle mass by the BIA method. This study was conducted in accordance with the Declaration of Helsinki and was approved by the ethics committee of the Jikei University School of Medicine (approval No. 28–196) and Fuji City General Hospital (approval No. 156).

### Diagnosis of sarcopenia, osteoporosis, and vertebral fracture

Sarcopenia was diagnosed using the JSH, AWGS, and EWGSOP2 criteria [1, 9, 10] (Additional file 1: Table S1). In the JSH criteria, sarcopenia is defined as having low handgrip strength (< 26 kg for males and < 18 kg for females) and low muscle mass (< 7.0 kg/m^2^ for males and < 5.7 kg/m^2^ for females) [1]. In the AWGS criteria, sarcopenia is defined as having low handgrip strength (< 26 kg for males and < 18 kg for females) and/or low gait speed (≤0.8 m/s both for males and females) and low muscle mass (< 7.0 kg/m^2^ for males and < 5.7 kg/m^2^ for females) [10]. In the EWGSOP2 criteria, sarcopenia is defined as having low handgrip strength (< 27 kg for males and < 16 kg for females) and low muscle mass (< 7.0 kg/m^2^ for males and < 5.5 kg/m^2^ for females). Low gait speed (≤0.8 m/s both for males and females) is an indicator for defining ‘severe sarcopenia’ [9]. Handgrip strength was assessed with a digital grip strength dynamometer (T.K. K5401 GRIP-D; Takei Scientific Instruments, Niigata, Japan). Muscle mass was assessed by the BIA method (InBody S10; InBody, Seoul, Korea). The SMI was calculated as the sum of the muscle mass of the four limbs divided by the height square (kg/m^2^). Gait speed was assessed over a distance of 6 m. BMD was assessed at the lumbar spine (L2-L4), femoral neck, and total hip using DEXA (PRODIGY; GE Healthcare, Madison, WI, USA). Osteoporosis was diagnosed according to the World Health Organization criteria (osteoporosis: T-score ≤ − 2.5; osteopenia: T-score between − 2.5 and − 1.0; normal: T-score > − 1.0) [18]. Vertebral fracture was evaluated using spinal lateral X-rays.

### Clinical and laboratory assessment

Blood samples were obtained from each patient after overnight fasting. The levels of serum albumin, total bilirubin, IGF-1, zinc, branched-chain amino acids (BCAAs), tartrate-resistant acid phosphatase (TRACP)-5b as a bone resorption marker, total procollagen type I N-terminal propeptide (P1NP) as a bone formation maker, 25-hydroxyvitamin D [25(OH)D], intact parathyroid hormone (PTH), and the prothrombin time-international normalized ratio (PT-INR) were measured by routine laboratory methods.

### Statistical analysis

Continuous variables were represented as medians, and 25th–75th interquartile ranges in parentheses. The Mann-Whitney U test and the Kruskal-Wallis test were used to evaluate the significance of differences in the distribution of continuous variables between the two groups and among the four groups, respectively. Categorical variables were represented as the number of patients, and percentages in parentheses. The chi-squared test was used with Cramér’s V, and adjusted residuals were calculated to evaluate the significance of differences in the distribution of categorical variables between groups. Kappa coefficients were calculated to evaluate the degree of agreement between the rates of sarcopenia assessed from each of the three different criteria. Univariate analysis was performed to evaluate possible variables that were significantly related to sarcopenia, osteoporosis, and vertebral fracture. Subsequently, multiple logistic regression analysis was performed to identify significant variables that were independently associated with these three complications. Correlations between two continuous variables were analyzed using the Spearman’s rank correlation test. The optimal cut-off values of SMI and handgrip strength required to estimate the presence or absence of osteoporosis were assessed by the area under the receiver operating characteristic (ROC) curves and were determined by the Youden index [19]. Statistical analyses were performed using SPSS version 25 (IBM, Armonk, NY, USA). A *P* value of < 0.05 was considered statistically significant.

## Results

### Patient characteristics

Among the 169 patients who were diagnosed with LC, 157 patients fulfilled the inclusion criteria; among these, 15 patients (8 with refractory ascites, 5 with metal in their body, and 2 undergoing hemodialysis) met the exclusion criteria and thus were excluded from this study. Therefore, 142 patients were finally included in the analysis. The baseline clinical characteristics and laboratory data of the 142 patients enrolled in this study are shown in Table 1. The patient cohort consisted of 90 males (63.4%) and 52 females (36.6%), with a median age of 70.5 (range, 58.8–76.0) years. The number of patients in each etiology group were as follows: hepatitis B virus (HBV) (*N* = 16), hepatitis C virus (HCV) (*N* = 45), alcohol (*N* = 48), and others (*N* = 33), which included primary biliary cholangitis (PBC), autoimmune hepatitis, nonalcoholic steatohepatitis, and cryptogenic CLD. The number of participants with Child-Pugh classes A and B/C was 94 and 48, respectively.

**Table 1:** Comparison of clinical characteristics between patients with and without sarcopenia

Variable	All patients	Sarcopenia	Non-sarcopenia	*P*-value
Patients, n (%)	142	48 (33.8)	94 (66.2)	
Age (years)	70.5 (58.8–76.0)	75.0 (71.3–79.8)	65.0 (54.8–73.2)	< 0.001
Male, n (%)	90 (63.4)	26 (54.2)	64 (68.1)	0.103
BMI (kg/m^2^)	23.7 (21.1–25.7)	21.3 (19.1–22.6)	24.5 (22.7–26.4)	< 0.001
Etiology				
HBV/HCV/Alcohol/others, n	16/45/48/33	3/25/11/9	13/20/37/24	0.003
Child-Pugh class A/B + C, n	94/48	32/16	63/31	0.996
Albumin (g/dL)	3.8 (3.4–4.3)	3.8 (3.3–4.3)	3.9 (3.4–4.2)	0.185
Total bilirubin (mg/dL)	0.9 (0.6–1.3)	0.8 (0.5–1.4)	0.9 (0.7–1.3)	0.170
Prothrombin time INR	1.11 (1.04–1.22)	1.10 (1.00–1.21)	1.13 (1.05–1.24)	0.193
IGF-1 (ng/mL)	54 (40–68)	46 (32–59)	59 (44–75)	< 0.001
Zinc (μg/dL)	65 (55–74)	64 (50–75)	65 (58–75)	0.280
BCAA (μmol/L)	395 (315–461)	319 (277–393)	421 (378–486)	< 0.001
TRACP-5b (mU/dL)	446 (344–589)	470 (368–629)	440 (326–575)	0.258
P1NP (ng/mL)	53 (35–80)	55 (34–80)	51 (36–80)	0.943
25(OH)D (ng/mL)	12.6 (9.1–16.8)	11.0 (8.9–15.3)	13.2 (9.2–17.6)	0.164
PTH (pg/mL)	44 (33–61)	50 (34–72)	43 (33–54)	0.168
SMI (kg/m^2^)				
All patients	6.77 (5.86–7.52)	5.56 (4.91–6.33)	7.18 (6.51–8.07)	< 0.001
Male	7.18 (6.68–8.07)	6.23 (5.61–6.80)	7.55 (7.13–8.32)	< 0.001
Female	5.83 (5.11–6.40)	5.13 (4.55–5.43)	6.32 (5.90–6.65)	< 0.001
Handgrip strength (kg)				
All patients	24.2 (17.2–32.6)	16.8 (14.1–22.8)	30.1 (22.2–36.8)	< 0.001
Male	30.2 (24.1–37.1)	22.1 (18.3–24.2)	34.3 (29.3–38.9)	< 0.001
Female	16.0 (14.0–21.4)	14.6 (12.6–15.8)	21.0 (15.0–22.8)	< 0.001
Gait speed under 0.8 (m/s), n (%)	42 (29.6)	26 (54.2)	16 (17.0)	< 0.001
Lumbar spine BMD (g/cm^2^)	1.08 (0.89–1.22)	0.95 (0.84–1.15)	1.12 (0.95–1.28)	< 0.001
Femoral neck BMD (g/cm^2^)	0.79 (0.65–0.89)	0.65 (0.59–0.78)	0.84 (0.72–0.93)	< 0.001
Total hip BMD (g/cm^2^)	0.83 (0.71–0.95)	0.71 (0.62–0.86)	0.87 (0.77–0.99)	< 0.001
Osteoporosis, n (%)	49 (34.5)	31 (64.6)	18 (19.1)	< 0.001
Vertebral fracture, n (%)	41 (28.9)	21 (43.8)	20 (21.3)	0.005

Values are shown as median (25th–75th interquartile range) or n (%). Statistical analysis was carried out using the chi-squared test or the Mann-Whitney U test, as appropriate. *BMI* Body mass index, *HBV* Hepatitis B virus, *HCV* Hepatitis C virus, *INR* International normalized ratio, *IGF-1* Insulin-like growth factor 1, *BCAA* Branched-chain amino acids, *TRACP-5b* Tartrate-resistant acid phosphatase 5b, *P1NP* Procollagen typeIN-terminal propeptide, *25(OH) D* 25-hydroxyvitamin D, *PTH* Parathyroid hormone, *SMI* Skeletal muscle mass index, *BMD* Bone mineral density

### Comparison of diagnostic outcomes of sarcopenia among the three different criteria

**Fig. 1:**
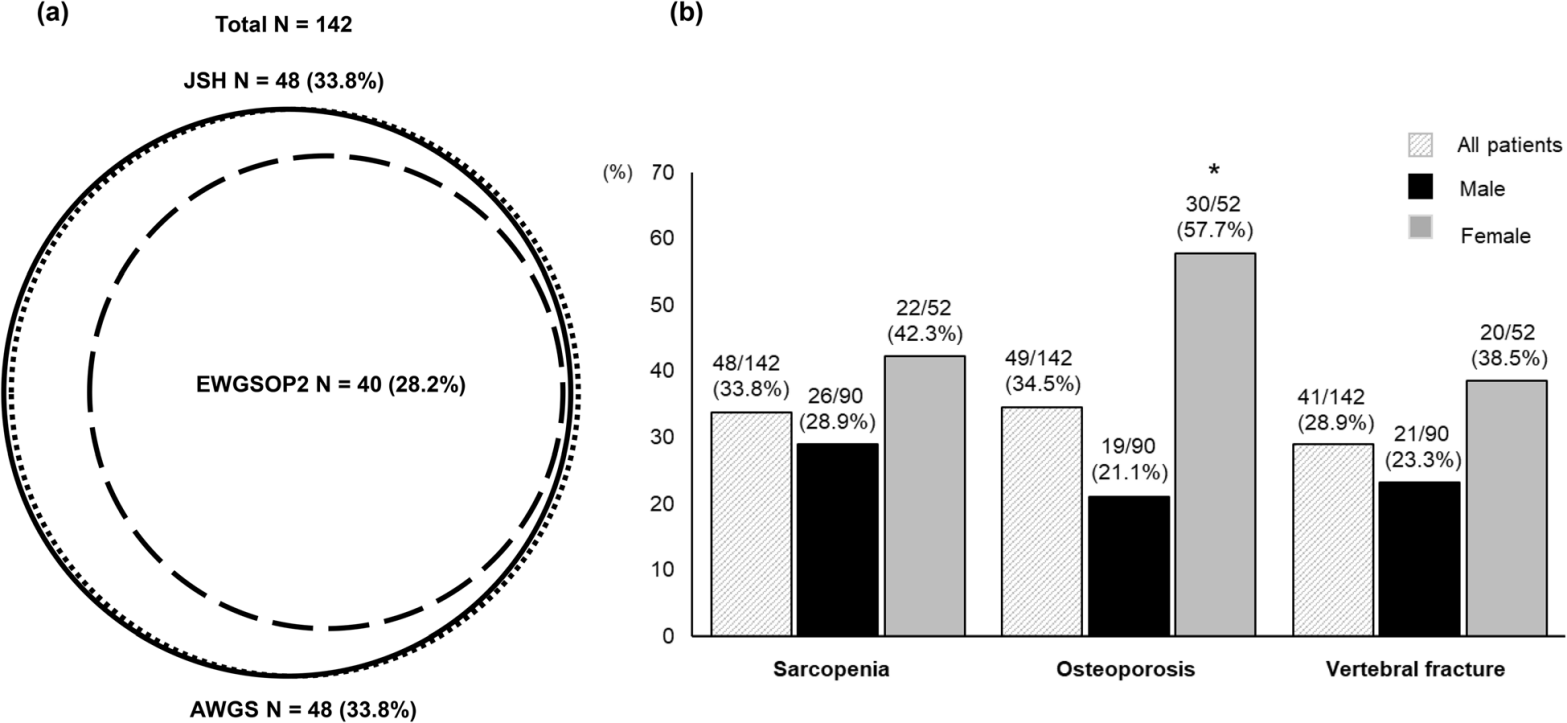
**a** Conformity of sarcopenia diagnosis as assessed using the JSH, AWGS, and EWGSOP2 criteria. The prevalence rate of sarcopenia was 33.8% (48/142) according to either the JSH or AWGS criteria, and 28.2% (40/142) according to the EWGSOP2 criteria. **b** Prevalence of sarcopenia, osteoporosis and vertebral fracture. Osteoporosis was more frequent in females than in males. * *P* < 0.001

The prevalence rate of sarcopenia was 33.8% (48/142) for all patients; 28.9% (26/90) for males and 42.3% (22/52) for females according to the JSH or AWGS criteria (Fig. 1a, b), whereas according to the EWGSOP2 criteria, they were 28.2% (40/142) for all patients; 28.9% (26/90) for males and 26.9% (14/52) for females. Patients diagnosed according to the JSH or AWGS criteria included all those diagnosed with sarcopenia by the EWGSOP2 criteria (Fig. 1a). All male sarcopenia patients fulfilled all the three criteria, whereas most female sarcopenia patients fulfilled both JSH and AWGS criteria, and eight female patients did not fulfill the EWGSOP2 criteria. According to the latter, the prevalence rate of severe sarcopenia was 57.5% (23/40). The kappa coefficients were 1.00 between the JSH and AWGS criteria and 0.87 between the JSH or AWGS and EWGSOP2 criteria. Taken together, the diagnostic outcome of the JSH criteria was identical to that of the AWGS criteria and similar to that of the EWGSOP2 criteria. Therefore, for this study, we presented sarcopenia-related results based on the JSH criteria.

### Comparison of clinical characteristics between patients with and without sarcopenia

Sarcopenia patients were older and had a lower body mass index (BMI) (*P* < 0.001 for both) than non-sarcopenia patients (Table 1). In the sarcopenia group, the most frequent etiology was HCV, whereas in the non-sarcopenia group, it was alcohol. Regarding the biochemical parameters, the levels of IGF-1 and BCAAs were significantly lower in the sarcopenia group than in the non-sarcopenia group (*P* < 0.001 for both).

All BMD values of the lumbar spine, femoral neck, and total hip in the sarcopenia group were significantly lower than those in the non-sarcopenia group (*P* < 0.001 for all). The prevalence rates of osteoporosis (64.6%) and vertebral fracture (43.8%) in the sarcopenia group were significantly higher compared to those in the non-sarcopenia group (19.1%, *P* < 0.001; and 21.3%, *P* = 0.005, respectively). These findings suggested that sarcopenia patients are more susceptible to osteoporosis and vertebral fracture than non-sarcopenia patients.

### Comparison of clinical characteristics between patients with and without osteoporosis

The prevalence rate of osteoporosis was 34.5% (49/142) for all patients; 21.1% (19/90) for males and 57.7% (30/52) for females (Fig. 1b); indicating that osteoporosis was more prevalent in females than in males (*P* < 0.001). Osteoporosis patients were older (*P* < 0.001) and had a lower BMI (*P* = 0.005) than non-osteoporosis patients (Table 2). The prevalence rates of HCV and compensated LC (Child-Pugh class A) were higher in the osteoporosis group than in the non-osteoporosis group (*P* = 0.009 and 0.020, respectively). Regarding the biochemical parameters, the osteoporosis group had significantly lower levels of total bilirubin, PT-INR, IGF-1, and BCAAs than the non-osteoporosis group.

**Table 2:** Comparison of clinical characteristics between with and without osteoporosis

Variable	Osteoporosis	Non-osteoporosis	P-value
Patients, n (%)	49 (34.5)	93 (65.5)	
Age (years)	76.0 (71.5–80.0)	65.0 (54.0–73.0)	< 0.001
Male, n (%)	19 (38.8)	71 (76.3)	< 0.001
BMI (kg/m^2^)	21.8 (19.6–24.9)	23.9 (22.3–26.1)	0.005
Etiology			
HBV/HCV/Alcohol/others, n	5/21/8/15	11/24/40/18	0.009
Child-Pugh class A/B + C, n	39/10	56/37	0.020
Albumin (g/dL)	4.0 (3.4–4.4)	3.8 (3.4–4.2)	0.391
Total bilirubin (mg/dL)	0.7 (0.5–1.0)	1.0 (0.7–1.4)	0.003
Prothrombin time INR	1.08 (1.00–1.14)	1.15 (1.06–1.27)	0.002
IGF-1 (ng/mL)	48 (41–62)	57 (40–77)	0.022
Zinc (μg/dL)	66 (55–76)	65 (55–74)	0.473
BCAA (μmol/L)	353 (294–457)	415 (336–471)	0.026
TRACP-5b (mU/dL)	460 (363–639)	444 (331–577)	0.371
P1NP (ng/mL)	49 (34–79)	54 (36–82)	0.599
25(OH)D (ng/mL)	12.7 (9.0–15.4)	12.5 (9.1–17.3)	0.593
PTH (pg/mL)	49 (36–70)	42 (32–56)	0.073
SMI (kg/m^2^)			
All patients	5.67 (5.00–6.47)	7.15 (6.46–8.06)	< 0.001
Male	6.41 (5.49–6.99)	7.38 (6.96–8.16)	< 0.001
Female	5.33 (4.82–5.92)	6.23 (5.80–6.59)	0.001
Handgrip strength (kg)			
All patients	16.8 (14.1–22.8)	30.1 (22.2–36.8)	< 0.001
Male	23.4 (18.3–25.9)	31.8 (27.3–38.8)	< 0.001
Female	14.7 (12.6–18.0)	21.1 (15.4–23.9)	0.002
Lumbar spine BMD (g/cm^2^)	0.86 (0.76–1.05)	1.17 (1.05–1.28)	< 0.001
Femoral neck BMD (g/cm^2^)	0.61 (0.55–0.65)	0.87 (0.79–0.94)	< 0.001
Total hip BMD (g/cm^2^)	0.65 (0.57–0.71)	0.91 (0.83–0.99)	< 0.001
Sarcopenia, n (%)	31 (63.3)	17 (18.3)	< 0.001
Vertebral fracture, n (%)	27 (55.1)	14 (15.1)	< 0.001

Values are shown as median (25th–75th interquartile range) or n (%). Statistical analysis was carried out using the chi-squared test or the Mann-Whitney U test, as appropriate. *BMI* Body mass index, *HBV* Hepatitis B virus, *HCV* Hepatitis C virus, *INR* International normalized ratio, *IGF-1* Insulin-like growth factor 1, *BCAA* Branched-chain amino acids, *TRACP-5b* Tartrate-resistant acid phosphatase 5b, *P1NP* Procollagen typeIN-terminal propeptide, *25(OH) D* 25-hydroxyvitamin D, *PTH* Parathyroid hormone, *SMI* Skeletal muscle mass index, *BMD* Bone mineral density

Among all patients (males and females), the SMI and handgrip strength values in the osteoporosis group were significantly lower than those in the non-osteoporosis group. The prevalence rates of sarcopenia (63.3%) and vertebral fracture (55.1%) in the osteoporosis group were significantly higher compared to those in the non-osteoporosis group (18.3%, *P* < 0.001; and 15.1%, *P* < 0.001, respectively). These findings suggested that osteoporosis patients suffer from sarcopenia and vertebral fracture at higher rates compared to non-osteoporosis patients.

### Comparison of clinical characteristics between patients with and without vertebral fracture

Among the 142 patients, 41 (28.9%) were diagnosed with vertebral fracture (Fig. 1b). The prevalence rates in males and females were 23.3% (21/90) and 38.5% (20/52), respectively. Intriguingly, among the 41 vertebral fracture patients, 24 (58.5%) developed vertebral fracture without symptoms. Vertebral fracture patients were older (*P* < 0.001) and had a lower BMI (*P* = 0.015), higher prevalence of compensated LC (*P* = 0.028), lower SMI (*P* < 0.001), lower handgrip strength (*P* = 0.002), and lower BMD (*P* < 0.001 for all) compared to non-vertebral fracture patients (Additional file 1: Table S2). The prevalence rates of sarcopenia (51.2%) and osteoporosis (65.9%) in the vertebral fracture group were significantly higher compared to those in the non-vertebral fracture group (26.7%, *P* = 0.005; and 21.8%, *P* < 0.001, respectively). These findings suggested that vertebral fracture patients have higher rates of both osteoporosis and sarcopenia.

### Factors associated with sarcopenia, osteoporosis, and vertebral fracture

We explored which variables were significantly and independently associated with sarcopenia, osteoporosis, and vertebral fracture.

First, the following five variables were significantly related to sarcopenia in the multivariate analysis: greater age [odds ratio (OR) = 1.084, 95% confidence interval (CI) = 1.028–1.142, *P* = 0.003], lower BMI (OR = 0.742, 95% CI = 0.615–0.894, *P* = 0.002), lower IGF-1 (OR = 0.962, 95% CI = 0.932–0.993, *P* < 0.001), lower BCAAs (OR = 0.990, 95% CI = 0.984–0.996, *P* = 0.001), and presence of osteoporosis (OR = 3.923, 95% CI = 1.381–11.140, *P* = 0.010) (Additional file 1: Table S3).

Next, the following four variables were significantly associated with osteoporosis in the multivariate analysis: greater age (OR = 1.081, 95% CI = 1.031–1.133, *P* = 0.001), male sex (OR = 0.130, 95% CI = 0.047–0.361, *P* < 0.001), presence of sarcopenia (OR = 5.722, 95% CI = 2.179–15.030, *P* < 0.001), and presence of vertebral fracture (OR = 4.615, 95% CI = 1.716–12.408; *P* = 0.002) (Additional file 1: Table S4).

Lastly, osteoporosis (OR = 6.838, 95% CI = 3.071–15.223, *P* < 0.001) was identified as the only variable associated with vertebral fracture in the multivariate analysis (Additional file 1: Table S5).

### Correlation between sarcopenia and osteoporosis in patients with LC

**Fig. 2:**
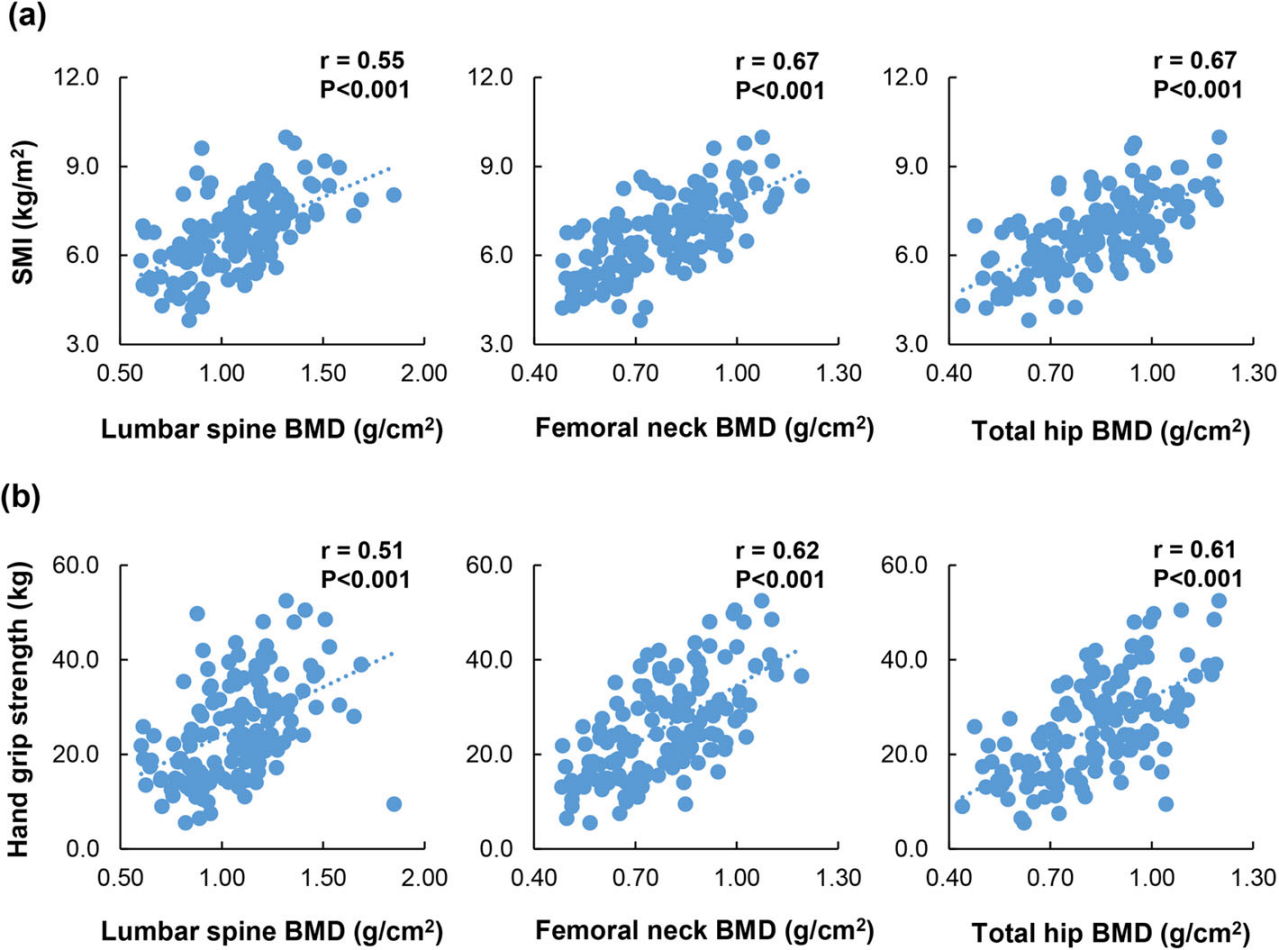
**a** Correlations between the SMI and BMD of the lumbar spine, femoral neck, and total hip in patients with liver cirrhosis. The SMI was significantly correlated with the BMD of the lumbar spine (*r* = 0.55, *P* < 0.001), femoral neck (*r* = 0.67, *P* < 0.001), and total hip (*r* = 0.67, *P* < 0.001). **b** Correlations between the handgrip strength and BMD of the lumbar spine, femoral neck, and total hip in patients with liver cirrhosis. The handgrip strength was significantly correlated with the BMD of the lumbar spine (*r* = 0.51, *P* < 0.001), femoral neck (*r* = 0.62, *P* < 0.001), and total hip (*r* = 0.61, *P* < 0.001)

**Fig. 3:**
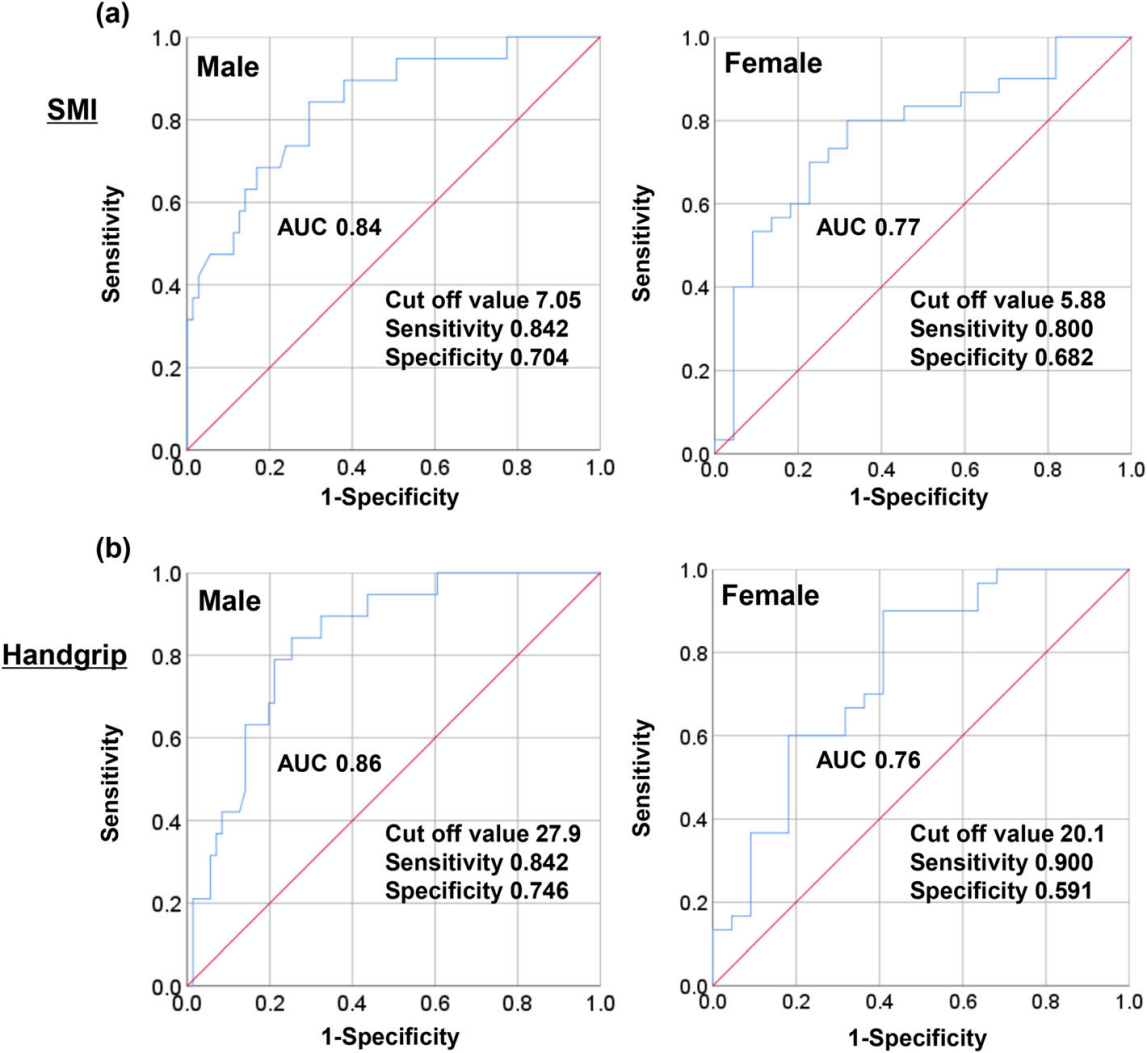
**a** The area under the receiver operating characteristic (ROC) curve of SMI for predicting osteoporosis in patients with liver cirrhosis. The SMI cut-off values (AUC, specificity, and sensitivity) were 7.05 kg/m^2^ (0.84, 0.842, and 0.704) for males, and 5.88 kg/m^2^ (0.77, 0.800, and 0.682, respectively) for females. **b** The ROC curve of handgrip strength for predicting osteoporosis in patients with liver cirrhosis. The handgrip strength cut-off values (AUC, specificity, and sensitivity) were 27.9 kg (0.84, 0.842, and 0.746, respectively) for males and 20.1 kg (0.76, 0.900 and 0.591, respectively) for females

As described above, there was a close relationship between sarcopenia and osteoporosis. Therefore, we evaluated the correlation between SMI, handgrip strength, and BMD (Fig. 2). There was a significant, positive correlation between SMI and BMD of the lumbar spine (*r* = 0.55); femoral neck (*r* = 0.67); and total hip (*r* = 0.67) (*P* < 0.001 for all). The handgrip strength was significantly correlated with the BMD of the lumbar spine, femoral neck, and total hip (*r* = 0.51, 0.62, and 0.61, respectively; *P* < 0.001 for all). In the ROC curve analysis (Fig. 3), the area under the curve (AUC) values for SMI were 0.84 in males and 0.77 in females. The SMI cut-off values for predicting osteoporosis were 7.05 kg/m^2^ for males and 5.88 kg/m^2^ for females, while the sensitivity was 0.842 for males and 0.800 for females, and the specificity was 0.704 for males and 0.682 for females. Similarly, the AUC values for handgrip strength were 0.86 in males and 0.76 in females. The optimal handgrip strength cut-off values, and its sensitivity and specificity, were 27.9 kg, 0.842, and 0.746 for males; and 20.1 kg, 0.900, and 0.591 for females, respectively.

**Fig. 4:**
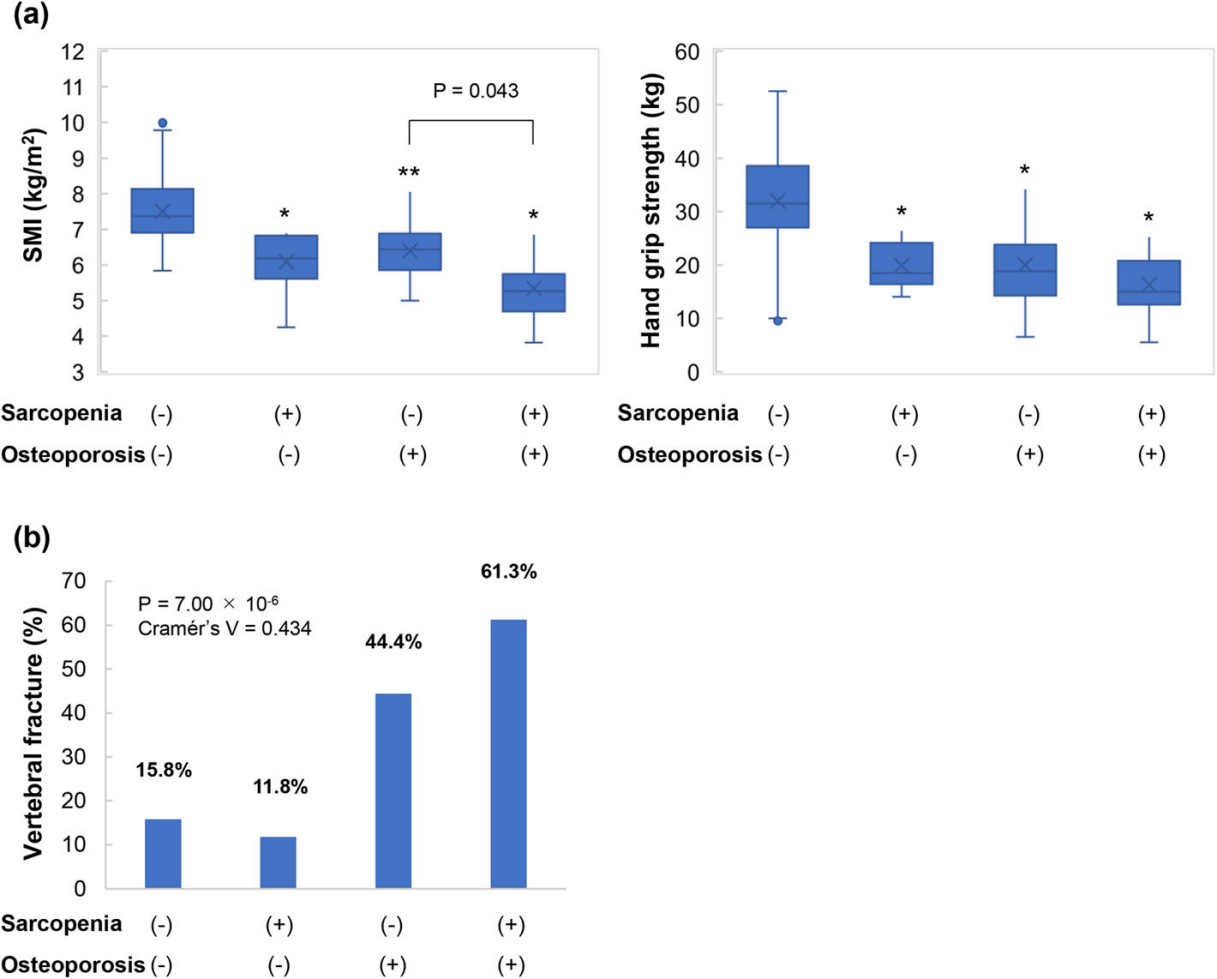
Comparison of clinical characteristics between four groups: (i) The sarcopenia (−)/osteoporosis (−) group, (ii) the sarcopenia (+)/osteoporosis (−) group, (iii) the sarcopenia (−) /osteoporosis (+) group, and (iv) the sarcopenia (+) /osteoporosis (+) (i.e., osteosarcopenia) group. **a** The SMI and handgrip strength values tended to be lowest in patients with both sarcopenia and osteoporosis. **b** The prevalence of vertebral fracture was significantly highest in the sarcopenia (+) /osteoporosis (+) (osteosarcopenia) group (adjusted residual = |4.5|), whereas it was significantly lowest in the sarcopenia (−) /osteoporosis (−) group (adjusted residual = |3.7|) (*P* = 7.00 × 10^− 6^; Cramér’s V = 0.434). * *P* < 0.001, ** *P* < 0.05 compared to the sarcopenia (−)/osteoporosis (−) group

Next, we divided the 142 patients into four groups: (i) patients without both sarcopenia and osteoporosis (76/142; 53.5%); (ii) patients with sarcopenia alone (17/142; 12.0%); (iii) patients with osteoporosis alone (18/142; 12.7%); and (iv) osteosarcopenia (31/142; 21.8%) (Additional file 2: Figure S1, Additional file 1: Table S6). In patients with osteosarcopenia, the values of SMI and handgrip strength tended to be the lowest among the four groups [Fig. 4a, Additional file 1: Table S6]. Notably, the prevalence rate of vertebral fracture (19/31; 61.3%) in patients with osteosarcopenia was significantly high among the four groups (adjusted residual = |4.5|), whereas the prevalence rate in patients without both sarcopenia and osteoporosis was significantly low among the four groups (adjusted residual = |3.7|) [Fig. 4b; *P* = 7.00 × 10^− 6^; Cramér’s V = 0.434].

## Discussion

In this study, we compared the prevalence of sarcopenia using three different diagnostic criteria. The cut-off values for low muscle mass in the JSH criteria were identical to those in the AWGS criteria and similar to those in the EWGSOP 2 criteria. Physical performance was included as an essential requirement in the AWGS criteria and used as an indicator of disease severity in the EWGSOP2 criteria, whereas it was omitted in the JSH criteria. We found that the prevalence of sarcopenia was 33.8% for both the JSH and AWGS criteria and 28.2% for the EWGSOP2 criteria. Notably, the diagnostic outcome using the JSH criteria was identical to that using the AWGS criteria. Furthermore, male patients diagnosed with sarcopenia were same using any of the three criteria. Among 42 patients with low physical performance diagnosed using the AWGS or EWGSOP2 criteria, 36 (85.7%) had low muscle strength and the remaining 6 (14.3%) did not show low muscle mass and thus did not reach a diagnosis of sarcopenia (data not shown). In future, these different diagnostic criteria should be harmonized to achieve more universal definitions.

Although each patient’s daily exercise was not fully quantified in the present study, most patients appeared to lack an exercise routine, and these circumstances may have increased the apparent prevalence of sarcopenia and osteoporosis. Moreover, as HCV infection was highly prevalent in the area (Fuji city) in the beginning of the 1900s, patients with HCV-related LC frequently reach an advanced age. In contrast, alcohol-induced LC is most common among newly diagnosed LC patients. Therefore, alcoholic patients were significantly younger than HCV patients (Additional file 1: Table S7). Furthermore, as a result of the rapid exacerbation of alcoholic liver dysfunction, decompensated LC developed more frequently in alcoholic patients than in HCV patients. In such situations, the present criteria proposed by the JSH have some limitations for diagnosing sarcopenia or evaluating disease severity in a heterogeneous population; these include younger patients with severe liver impairment and older patients with less severe liver impairment. Therefore, revisions of the diagnostic criteria, such as stratification by age, disease stage, and/or etiology, are necessary for the accurate diagnosis and assessment of sarcopenia.

In the present study, decreased levels of BCAAs and IGF-1 were significant independent factors associated with sarcopenia. Branched-chain amino acids, consisting of leucine, isoleucine, and valine, are essential for increasing and maintaining muscle mass [20, 21]. As the liver is a multifunctional organ involved in carbohydrate, protein, and lipid metabolism, LC is complicated by PEM and hyperammonemia and can lead to the consumption of BCAAs by skeletal muscles for energy production and ammonia metabolism [22]. IGF-1, produced by hepatocytes and myocytes, is involved in muscle protein synthesis, and the mammalian target of rapamycin (mTOR), activated by protein kinase B (PCK/AKT), stimulates protein synthesis [20]. The mTOR pathway is activated by IGF-1, BCAAs (particularly leucine), and exercise. The proliferation of satellite cells, which are the precursors of new muscle fibers, is essential for muscle growth. Satellite cell activation is stimulated by protein kinase B and promoted by IGF-1 and BCAAs. On the other hand, the proliferation of satellite cells is suppressed by myostatin, which is a cytokine belonging to the transforming growth factor-β family. The IGF-1 signaling pathway inhibits myostatin and stimulates muscle growth. Reportedly, higher myostatin levels correlate with a loss of muscle mass and reduced survival in patients with LC [23]. Taken together, our results are theoretically reasonable and support the notion that decreased levels of both BCAA and IGF-1 are associated with the development and progression of sarcopenia in patients with LC.

Since osteoporosis is one of the most common complications of LC, the term ‘hepatic osteodystrophy’ is often used to describe bone disorders in subjects with CLD. According to the WHO criteria, the prevalence rates of osteoporosis, osteopenia, and normal BMD in the present study were 34.5, 40.1, and 25.4%, respectively (data not shown). Although the pathogenesis of osteoporosis is not entirely understood, an imbalance in bone remodeling is known to affect the development of bone loss in CLD. IGF-1 is essential for bone remodeling and the maintenance of bone mass and strength due to its stimulation of osteoblast differentiation and proliferation, and its regulation of diaphyseal growth [24, 25]. The present study also showed that low IGF-1 levels were associated with osteoporosis, although there was no statistical significance in the multivariate analysis.

The prevalence of Child-Pugh class B/C (decompensated LC) and the values of total bilirubin and PT-INR were higher in non-osteoporosis patients than in osteoporosis patients. One possible explanation for these paradoxical results is that the etiology components differed between the two groups. Hepatitis C virus was the most frequent etiology (42.9%) in osteoporosis patients, whereas alcohol was the most frequent etiology (43.0%) in non-osteoporosis patients. As described above, alcoholic patients were significantly younger than HCV patients and more frequently developed decompensated LC. As the total bilirubin and PT-INR values were significantly higher in alcoholic patients than in HCV patients, a higher frequency of alcoholic etiology may cause higher total bilirubin and PT-INR values in non-osteoporosis patients.

Sarcopenia and rapid skeletal muscle wasting are associated with mortality and reduced QOL in patients with LC [8, 26, 27]. Osteoporosis predisposes patients to fragility fractures, which affect both morbidity and QOL, and the early diagnosis and treatment of sarcopenia and osteoporosis are important. Recently, the term ‘osteosarcopenia’ was defined when sarcopenia and osteoporosis coexist [14, 15]. Reportedly, the prevalence of osteosarcopenia was 28.7% in patients aged ≥60 years with hip fractures, and the 1-year mortality in osteosarcopenia patients was higher than that in other groups: normal, 7.8%; osteoporosis only, 5.1%; and sarcopenia only, 10.3% [28]. A study on community-dwelling Chinese elders showed that the prevalence of osteosarcopenia was 10.4% in males and 15.1% in females [29], and that patients with osteosarcopenia were more susceptible to fragility fractures, frailty, and mortality [28–30]. In our study, the prevalence of osteosarcopenia was 21.8% (31/142) for all patients; 15.6% (14/90) for males and 32.7% (17/52) for females. The prevalence of vertebral fractures in osteosarcopenia patients was 61.3% (31/19), which was the highest among the four patient groups. Thus, osteosarcopenia may increase the frequency of vertebral fractures. Importantly, more than half (24/41; 58.5%) of the patients with vertebral fracture were newly diagnosed in the present study; these patients were either asymptomatic or had no opportunity to evaluate the vertebral fracture using spinal X-rays. Therefore, we should carefully interview patients regarding fracture-related symptoms and assess vertebral fracture using radiological imaging tests, especially in LC patients with osteosarcopenia.

Regarding the association between muscle and bone, recent reports have shown that sarcopenia is independently associated with low BMD in patients with CLD [31, 32].

In the present study, the SMI and handgrip strength values were significantly correlated with the BMD of the lumbar spine, femoral neck, and total hip. In the ROC curve analysis, the SMI cut-off values for predicting osteoporosis were 7.05 kg/m^2^ in males and 5.88 kg/m^2^ in females. Similarly, the handgrip strength cut-off values for predicting osteoporosis were 27.9 kg in males and 20.1 kg in females. Intriguingly, these cut-off values almost coincided with those for the sarcopenia diagnostic criteria, which were proposed by the JSH, AWGS, and EWGSOP2. These findings suggest that a diagnosis of sarcopenia may be useful for predicting the presence of osteoporosis in patients with LC.

Recently, the EWGSOP proposed the concept of malnutrition-associated sarcopenia whereby a sarcopenia phenotype is related to malnutrition irrespective of the cause (reduced food intake, low nutrient bioavailability, or high nutrient requirements including inflammatory diseases such as CLD and malignancy) [9, 33, 34]. The definition of malnutrition by the Global Leadership Initiative on Malnutrition (GLIM) recommends low muscle mass as one of its criterion items [9, 34]. Hence, low muscle mass is a common feature in both malnutrition and sarcopenia. Reportedly, patients with sarcopenia showed an increased risk of malnutrition, and conversely, hospitalized older patients with malnutrition were more susceptible to sarcopenia [35]. As the liver is an organ central to nutrient metabolism, malnutrition and sarcopenia are frequently observed and closely linked together in patients with LC. Therefore, assessment of the nutrition status and malnutrition risk is important to ascertain the causes of loss muscle mass and its pathological mechanisms.

Branched-chain amino acid supplementation stimulates albumin and protein synthesis in skeletal muscle and has the potential to improve the prognosis of LC patients with sarcopenia [8, 21]. In particular, the administration of leucine-enriched foods activates the mTOR pathway and increases muscle protein synthesis [36]. Reportedly, decreased physical activity and insufficient energy intake are associated with sarcopenia in patients with LC, regardless of disease progression [37]. Therefore, exercise regimens involving walking ≥5000 steps/day with a total energy intake of approximately 30 kcal/ideal body weight are recommended [37]. In another report, moderate physical exercise together with leucine supplements improved exercise capacity, leg muscle mass, and health-related QOL [38]. Regarding the pharmacological treatment of osteoporosis, weekly alendronate or monthly ibandronate has been shown to increase bone mass in PBC patients. Cyclical etidronate, a bisphosphonate with an antiresorptive effect, increases bone mass in female patients with LC [39]. However, the majority of previous studies only included a small number of patients and predominantly targeted PBC patients or transplant recipients [40–42]. Thus, the research regarding osteoporosis treatment for patients with LC has not reached a definite conclusion. A large-scale trial for new drugs is needed in order to establish the treatment for osteoporosis in patients with LC.

This study had some limitations. First, we used the BIA method for the assessment of muscle mass, as per the JSH criteria recommendation (as well as CT). BIA equipment, although safe, non-invasive, and easy to use, does not measure muscle mass directly and is sensitive to patients’ conditions, such as hydration and ascites [43]. In addition, the CT method needs proprietary software for analysis. Second, this study did not include a nutritional intake assessment. In future, conducting a nutritional intake assessment by an expert nutritionist will aid in the investigation of the relationship between malnutrition and sarcopenia.

## Conclusions

A high prevalence of sarcopenia diagnosed by three different criteria, osteoporosis, osteosarcopenia, and vertebral fracture, was found in patients with LC, and the present study showed a close association between these clinical events. The SMI and handgrip strength values were significantly correlated with BMD and may be useful for the diagnosis of osteoporosis. Patients with osteosarcopenia showed the highest risk of vertebral fractures and considerable attention for asymptomatic vertebral fractures is necessary particularly in patients with LC. This study may provide a useful reference for the occurrence of sarcopenia and osteoporosis in patients with LC. Further investigation may provide a future therapeutic strategy for skeletal muscle and bone disorders to improve the QOL and prognosis of patients with LC.

## Supplementary information

**Additional file 1: Table S1.** The cut-off values for handgrip, SMI, and gait speed in JSH, AWGS, and EWGSOP2 criteria. **Table S2.** Comparison of clinical characteristics between patients with and without vertebral fracture. **Table S3.** Univariate and multivariate analyses of variables associated with sarcopenia in patients with liver cirrhosis. **Table S4.** Univariate and multivariate analyses of variables associated with osteoporosis in patients with liver cirrhosis. **Table S5.** Univariate and multivariate analyses of variables associated with vertebral fracture in patients with liver cirrhosis. **Table S6.** Characteristics of patients with and without sarcopenia/osteoporosis. **Table S7.** Comparison of clinical characteristics by etiology.

**Additional file 2: Figure S1.** Relationship between sarcopenia and osteoporosis in patients with liver cirrhosis.

## Data Availability

The data collected and analyzed in the current study are available from the corresponding authors on reasonable request.

## Abbreviations

25(OH) D: 25-hydroxyvitamin D
BCAA: Branched-chain amino acids
BIA: Bioelectrical impedance analysis
BMD: Bone mineral density
BMI: Body mass index
CI: Confidence interval
CLD: Chronic liver disease
DEXA: Dual-energy X-ray absorptiometry
HBV: Hepatitis B virus
HCV: Hepatitis C virus
IGF-1: Insulin-like growth factor 1
INR: International normalized ratio
LC: Liver cirrhosis
OR: Odds ratio
P1NP: Procollagen type I N-terminal propeptide
PTH: Parathyroid hormone
ROC: Receiver operating characteristic
SMI: Skeletal muscle mass index
TRACP-5b: Tartrate-resistant acid phosphatase 5b
