# Supplementary material for: Comparative assessment of sarcopenia using the JSH, AWGS, and EWGSOP2 criteria and the relationship between sarcopenia, osteoporosis, and osteosarcopenia in patients with liver cirrhosis
Source: BMC Musculoskelet Disord. 2019 Dec 26;20:615. doi: 10.1186/s12891-019-2983-4 (PMC6933666; doi:10.1186/s12891-019-2983-4)
Supplement: Supplementary file 1 — Additional file 1: Table S1. The cut-off values for handgrip, SMI, and gait speed in JSH, AWGS, and EWGSOP2 criteria. Table S2. Comparison of clinical characteristics between patients with and without vertebral fracture. Table S3. Univariate and multivariate analyses of variables associated with sarcopenia in patients with liver cirrhosis. Table S4. Univariate and multivariate analyses of variables associated with osteoporosis in patients with liver cirrhosis. Table S5. Univariate and multivariate analyses of variables associated with vertebral fracture in patients with liver cirrhosis. Table S6. Characteristics of patients with and without sarcopenia/osteoporosis. Table S7. Comparison of clinical characteristics by etiology. [file 12891_2019_2983_MOESM1_ESM.docx]

**Table S1 The cut-off values for handgrip, SMI, and gait speed in JSH, AWGS, and EWGSOP2 criteria**

JSH, Japan Society of Hepatology; AWGS, Asian Working Group for Sarcopenia; EWGSOP, European Working Group on Sarcopenia in Older People.

**Table S2 Comparison of clinical characteristics between patients with and without vertebral fracture**

Values are shown as median (25th–75th interquartile range) or n (%). Statistical analysis was carried out using the chi-squared test or the Mann-Whitney U test, as appropriate. BMI, body mass index; HBV, hepatitis B virus; HCV, hepatitis C virus; INR, international normalized ratio; IGF-1, insulin-like growth factor 1; BCAA, branched-chain amino acids; TRACP-5b, tartrate-resistant acid phosphatase 5b; P1NP, procollagen typeⅠN-terminal propeptide; 25(OH)D, 25-hydroxyvitamin D; PTH, parathyroid hormone; SMI, skeletal muscle mass index; BMD, bone mineral density.

**Table S3 Univariate and multivariate analyses of variables associated with sarcopenia in patients with liver cirrhosis**

BMI, body mass index; HBV, hepatitis B virus; HCV, hepatitis C virus; INR, international normalized ratio; IGF-1, insulin-like growth factor 1; BCAA, branched-chain amino acids; TRACP-5b, tartrate-resistant acid phosphatase 5b; P1NP, procollagen typeⅠN-terminal propeptide; 25(OH)D, 25-hydroxyvitamin D; PTH, parathyroid hormone; SMI, skeletal muscle mass index; BMD, bone mineral density; OR, odds ratio; CI, confidence interval.

**Table S4 Univariate and multivariate analyses of variables associated with osteoporosis in patients with liver cirrhosis**

BMI, body mass index; HBV, hepatitis B virus; HCV, hepatitis C virus; INR, international normalized ratio; IGF-1, insulin-like growth factor 1; BCAA, branched-chain amino acids; TRACP-5b, tartrate-resistant acid phosphatase 5b; P1NP, procollagen typeⅠN-terminal propeptide; 25(OH)D, 25-hydroxyvitamin D; PTH, parathyroid hormone; SMI, skeletal muscle mass index; BMD, bone mineral density; OR, odds ratio; CI, confidence interval.

**Table S5 Univariate and multivariate analyses of variables associated with vertebral fracture in patients with liver cirrhosis**

BMI, body mass index; HBV, hepatitis B virus; HCV, hepatitis C virus; INR, international normalized ratio; IGF-1, insulin-like growth factor 1; BCAA, branched-chain amino acids; TRACP-5b, tartrate-resistant acid phosphatase 5b; P1NP, procollagen typeⅠN-terminal propeptide; 25(OH)D, 25-hydroxyvitamin D; PTH, parathyroid hormone; SMI, skeletal muscle mass index; BMD, bone mineral density; OR, odds ratio; CI, confidence interval.

**Table S6 Characteristics of patients with and without sarcopenia/osteoporosis**

Values are shown as median (25th–75th interquartile range) or n (%). Statistical analysis was carried out using the chi-squared test or the Kruskal-Wallis test, as appropriate. BMI, body mass index; HBV, hepatitis B virus; HCV, hepatitis C virus; INR, international normalized ratio; IGF-1, insulin-like growth factor 1; BCAA, branched-chain amino acids; TRACP-5b, tartrate-resistant acid phosphatase 5b; P1NP, procollagen typeⅠN-terminal propeptide; 25(OH)D, 25-hydroxyvitamin D; PTH, parathyroid hormone; SMI, skeletal muscle mass index; BMD, bone mineral density.

**Table S7 Comparison of clinical characteristics by etiology**

****Values are shown as median (25th–75th interquartile range) or n (%). Statistical analysis was carried out using the chi-squared test or the Kruskal-Wallis-test, as appropriate. INR, international normalized ratio.
