# Supplementary figures and images for: Comparative assessment of sarcopenia using the JSH, AWGS, and EWGSOP2 criteria and the relationship between sarcopenia, osteoporosis, and osteosarcopenia in patients with liver cirrhosis
Source: BMC Musculoskelet Disord. 2019 Dec 26;20:615. doi: 10.1186/s12891-019-2983-4 (PMC6933666; doi:10.1186/s12891-019-2983-4)

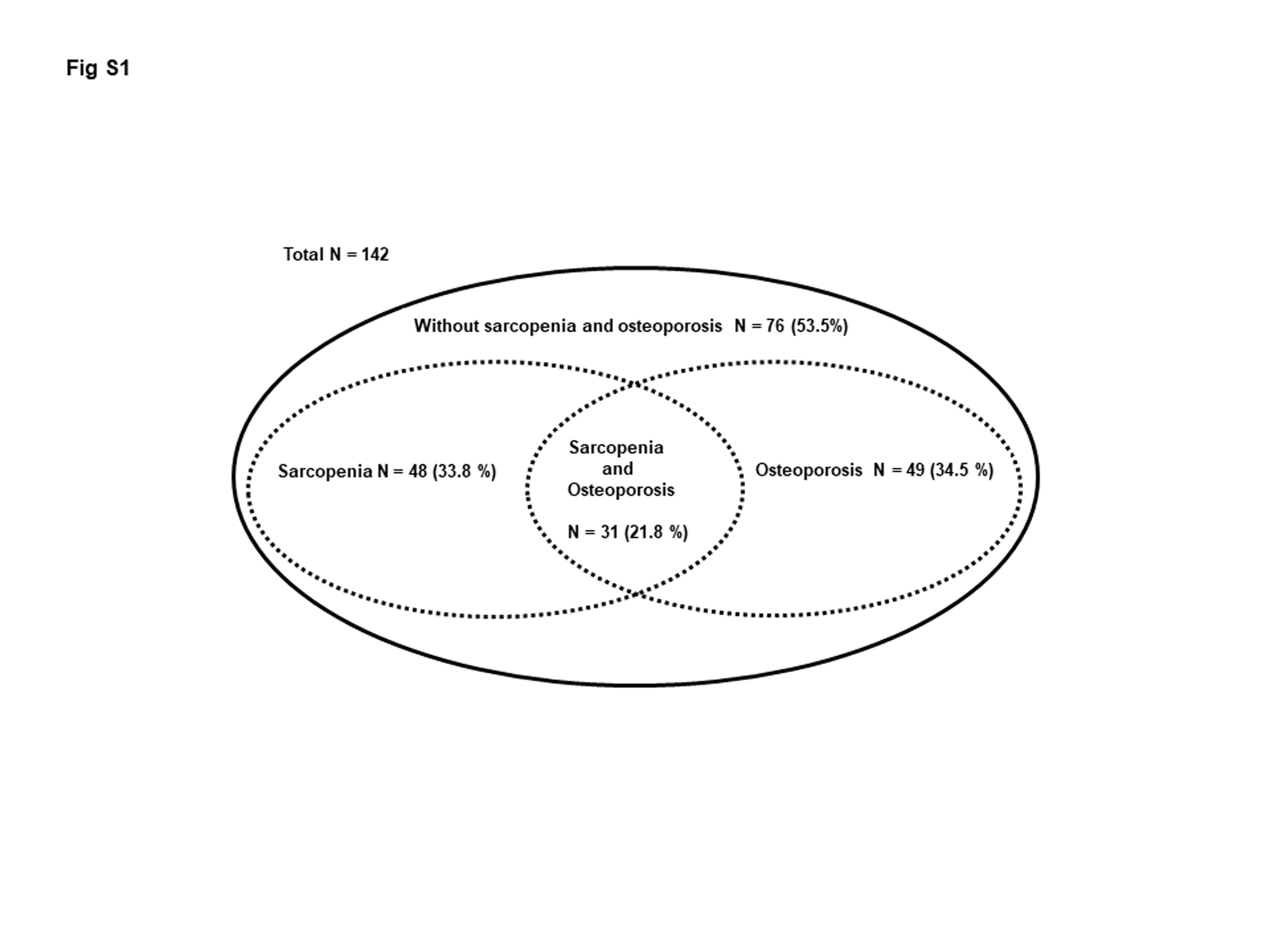

Supplement: Supplementary file 2 — Additional file 2: Figure S1. Relationship between sarcopenia and osteoporosis in patients with liver cirrhosis. [file 12891_2019_2983_MOESM2_ESM.tif]
